# Larval Zebrafish as a Model for Mechanistic Discovery in Mental Health

**DOI:** 10.3389/fnmol.2022.900213

**Published:** 2022-06-24

**Authors:** Jazlynn Xiu Min Tan, Ryan Jun Wen Ang, Caroline Lei Wee

**Affiliations:** Institute of Molecular and Cell Biology, Agency for Science, Technology and Research (A*STAR), Singapore, Singapore

**Keywords:** zebrafish, stress, anxiety, depression, mental health, endophenotype, neuropsychiatric disorders

## Abstract

Animal models are essential for the discovery of mechanisms and treatments for neuropsychiatric disorders. However, complex mental health disorders such as depression and anxiety are difficult to fully recapitulate in these models. Borrowing from the field of psychiatric genetics, we reiterate the framework of ‘endophenotypes’ – biological or behavioral markers with cellular, molecular or genetic underpinnings – to reduce complex disorders into measurable behaviors that can be compared across organisms. Zebrafish are popular disease models due to the conserved genetic, physiological and anatomical pathways between zebrafish and humans. Adult zebrafish, which display more sophisticated behaviors and cognition, have long been used to model psychiatric disorders. However, larvae (up to 1 month old) are more numerous and also optically transparent, and hence are particularly suited for high-throughput screening and brain-wide neural circuit imaging. A number of behavioral assays have been developed to quantify neuropsychiatric phenomena in larval zebrafish. Here, we will review these assays and the current knowledge regarding the underlying mechanisms of their behavioral readouts. We will also discuss the existing evidence linking larval zebrafish behavior to specific human behavioral traits and how the endophenotype framework can be applied. Importantly, many of the endophenotypes we review do not solely define a diseased state but could manifest as a spectrum across the general population. As such, we make the case for larval zebrafish as a promising model for extending our understanding of population mental health, and for identifying novel therapeutics and interventions with broad impact.

## Population Mental Health and Endophenotypes

Mental health disorder prevalence has increased globally, and has recently been aggravated by the onset of the COVID-19 pandemic, with an estimated worldwide increase of 682.4 cases of major depressive disorder and 977.5 cases of anxiety disorders per 100,000 population in 2020 alone (Santomauro et al., 2021). Depression and anxiety arising due to COVID-19 are estimated to have caused 49.4 and 44.5 million Disability adjusted life years (DALYs), respectively, the metric used by the biennial global burden of disease study to represent the number of years of healthy life lost to disability or death caused by disease (Santomauro et al., 2021).

Even though the Diagnostic and Statistical Manual of Mental Disorders (DSM) (American Psychiatric Association, 2013) has clear-cut criteria for mental illness diagnosis, there is also increasing evidence that mental health exists as a spectrum, or continuum of states, rather than a binary classification of healthy vs. ill (Keyes, 2002; Kupfer and Regier, 2011). Multiple disorders share common risk factors, and disorders arise through a combination of gene effects and environmental factors. Hence, Hyman (2010) summarizes the arguments for a mental health continuum model, highlighting the current limitations of a categorical system.

Despite the prevalence and severity of mental illnesses or ill-being, the development of interventions has lagged behind. We attribute this to at least two reasons. Firstly, mental health disorders are highly complex and heterogeneous, involving multiple interacting mechanisms. Secondly, no animal model can fully recapitulate the intricacies of these disorders, leaving us limited in studying disease mechanisms and searching for potential therapeutics. Rather than tackling such a multidimensional problem head on, it may be helpful to focus on individual quantifiable features of such disorders, which could then be dissected in diverse model systems. Hence, the endophenotypic approach is an attractive solution, wherein complex disorders are dissected into simpler components (“endophenotypes”).

The term “endophenotype” was first used in the field of psychiatric genetics by Gottesman and Shields (1967, 1973) to discuss schizophrenia, and was further refined by Gottesman and Gould (2003). According to their definition, an endophenotype must be associated with a disease, heritable, independent of disease state, cosegregate with the disease within families and found in unaffected relatives more than the general population. Different patients, even under the same diagnosis, may present with distinct endophenotypes (Chen et al., 2000; Nandi et al., 2009; Lee et al., 2016; Buch and Liston, 2021), hence dissecting a complex disorder into these individual components can also allow for more personalized and targeted treatments for their illness.

An example of an endophenotype is prepulse inhibition (PPI), which reflects compromised sensorimotor gating in schizophrenia (Parwani et al., 2000), as well as in obsessive-compulsive disorder and Huntington’s disease (Swerdlow et al., 1995; Hoenig et al., 2005). Healthy individuals have a startle response when presented with a strong sensory stimulus, which can be attenuated if a weaker stimulus precedes it by around 100 ms – this attenuation is not observed in patients with these disorders. Genome-wide association studies identified a strong correlation between the AKT3 gene locus and schizophrenia (Ripke et al., 2013), where patients’ brains had lower expression levels of AKT3 protein (van Beveren et al., 2012). In zebrafish, PPI defects were similarly found in *akt3* mutants (Thyme et al., 2019). Since sensorimotor gating impairment via prepulse inhibition has a genetic underpinning and segregates schizophrenia patients from healthy individuals, it is a strong endophenotype, which shares conserved mechanisms across species including zebrafish.

Whereas schizophrenia only affects a small subset of the population, other mental health disorders such as depression and anxiety exist as a broad spectrum (Angst et al., 2000; Lang and McTeague, 2009; Dichter et al., 2012; McTeague and Lang, 2012) and reflect dysregulation of motivated behavioral drives that are deeply conserved across evolution (LeDoux, 2012, 2021). In this review, we will more broadly define endophenotypes as behavioral markers that are associated with a human disorder and have a neural circuit or molecular-genetic underpinning (Table 1). We will identify parallel behaviors in larval zebrafish and compare the extent to which they can recapitulate human endophenotypes and their underlying mechanisms. Importantly, the identified behaviors can be assayed in a high-throughput manner using this model, facilitating both drug screening and mechanistic dissection which are key for discovering treatments for mental health disorders and improving mental health across the population.

**TABLE 1:** Definitions.

Term	Definition	References
Endophenotype	Behavioral markers that are associated with a human disorder and have a neural circuit or molecular-genetic underpinning.	Gottesman and Shields, 1967, 1973; Gottesman and Gould, 2003
Stress	Physiological/hormonal response to disruption of homeostasis (caused by threat or environmental changes e.g., salinity).	Munck et al., 1984; Chrousos, 2009; Tsigos et al., 2020
Anxiety	Brain state induced by exposure to threatening or dangerous stress(ors), that persists even after the removal of danger/threat, in anticipation of future threat, and which is reflected by behavioral changes such as an enhancement of defensive behavior. A diagnosis of anxiety disorder is made when individuals display persistent and excessive prolonged anxiety with related behavioral disturbances.	Rosen and Schulkin, 1998; Martin et al., 2010; American Psychiatric Association, 2013; Lang et al., 2014
Fear	Acute brain state induced by external threat that triggers defensive behavior (fight, flight, and freeze).	Rosen and Schulkin, 1998; Misslin, 2003; American Psychiatric Association, 2013
Depression	Brain state reflected by reduced motivation to perform normal survival behaviors (e.g., feeding/defense), and anhedonia. May be induced by inescapable threat or other factors. A diagnosis of depressive disorders is made when this dysregulated emotional/motivational state (i.e., mood) is accompanied by behavioral or physiological changes that impair normal function.	Pizzagalli et al., 2008; Admon and Pizzagalli, 2015; Proulx et al., 2018
Defensive behaviors	Defensive behaviors arise as a survival response to threats, and include behaviors like freezing, escape, or avoidance. This is opposed to aggression where the threat is actively engaged.	Blanchard et al., 1986; Blanchard, 1997; Sternson, 2013; Lovett-Barron et al., 2020
Avoidance behaviors	The act of avoiding or moving away from a negatively valenced situation or environment. These behaviors are often exaggerated in high-stress conditions. Occasionally used interchangeably with “defensive behaviors,” particularly in reference to escape maneuvers.	Bandura and Menlove, 1968; Russell and Mehrabian, 1978; Chen and Bargh, 1999; Elliot, 2013
Motivated behaviors	There are behaviors that are energized by the motivation to survive. Deficits in motivation are observed in multiple psychiatric disorders including anxiety and depression.	Simpson and Balsam, 2016
Appetitive behaviors	Appetitive behaviors are behaviors executed to seek out rewarding stimuli.	Hoebel, 1997; Ball and Balthazart, 2008

## Advantages of Larval Zebrafish

Zebrafish are popular models in studying neurological phenomena as summarized by Kalueff et al. (2014a) and Stewart et al. (2014). Sharing 70% of their genes with humans and conserved physiological and neuronal pathways (Howe et al., 2013; Stewart et al., 2015), many neurobehavioral parallels have been uncovered between zebrafish and mammals, including humans. For example, both the human and zebrafish hypothalamic-pituitary-adrenal (HPA) axes [also known as the hypothalamic-pituitary-interrenal (HPI) axis in zebrafish] are activated in response to stress, leading to the secretion of corticotropin releasing hormone/factor (CRH/CRF) which stimulates pituitary corticotroph cells to release adrenocorticotropic hormone (ACTH). ACTH then activates the adrenal glands to produce and release cortisol into the blood. Unlike in rodent models, where corticosterone is the main stress hormone (Joëls et al., 2018), cortisol is the main stress hormone in both fish and humans, and exerts wide-ranging effects on physiology to allow the body to cope with stress. Depression and anxiety, amongst other mental health disorders, have been associated with HPA anomalies and cortisol dysregulation (Feder et al., 2004; Erhardt et al., 2006; Faravelli et al., 2012; Nandam et al., 2019). Hence, the zebrafish shares a core feature of human stress biology.

Not surprisingly, zebrafish have been used extensively to model psychiatric disorders and identify pharmacological interventions. Many such studies and reviews have focused on adult zebrafish (Cachat et al., 2010; Maximino et al., 2018) as they have more complex behaviors and a fully-developed brain. Nevertheless, there are significant benefits to using larvae, such as increased scalability and tractability. At the larval stage (up to 30 days old), zebrafish are numerous, small, and optically transparent. Larvae only require small volumes of water and can be contained in 48- or 96-well plates. A top view camera can be used to acquire videos of many larvae simultaneously during behavioral assays for live or post-acquisition analysis. This makes it well-suited for drug screening which emphasizes throughput. Beyond drug screening, dietary interventions (O’Neil et al., 2014; Godos et al., 2020) and the role of probiotics (Liu, 2017; Skonieczna-Żydecka et al., 2018) can also be explored. Furthermore, brain imaging to study the underlying biological mechanisms of endophenotypes or interventions is more convenient due to their transparency. Hence, this review will focus primarily on the potential of larval zebrafish to model human endophenotypes in mental health and disease.

## Human and Zebrafish Anxiety and Mood Endophenotypes

While there are many mental health disorders, we will primarily be discussing anxiety/stress-related and mood disorders (see Table 1 for definitions). The Diagnostic and Statistical Manual (DSM5) details a specific set of criteria for diagnosis of these disorders based on symptoms and behaviors exhibited (American Psychiatric Association, 2013). Anxiety is a brain state induced by dangerous or stressful situations that continues to persist even after the threat has subsided (Rosen and Schulkin, 1998; Martin et al., 2010; Lang et al., 2014). Anxiety and stress-related disorders are diagnosed when the individual experiences persistent and excessive fear and anxiety and related behavioral disturbances (American Psychiatric Association, 2013). These are accompanied by physical symptoms such as restlessness, fatigue, difficulty concentrating, irritability and sleep disturbances (American Psychiatric Association, 2013). Depressive disorders are part of a broader class of mood disorders in which emotional and motivational states are dysregulated. In depression, individuals experience persistent feelings of sadness, irritability, emptiness, or anhedonia as well as a reduction in ability and motivation to perform normal survival functions (Pizzagalli et al., 2008; Admon and Pizzagalli, 2015; Proulx et al., 2018). Diagnostic criteria include exhibiting some subset of symptoms that include diminished interest in daily activities, insomnia, appetite or weight gain or loss, psychomotor agitation or retardation, fatigue, feelings of guilt or suicidal ideation (American Psychiatric Association, 2013).

**FIGURE 1:**
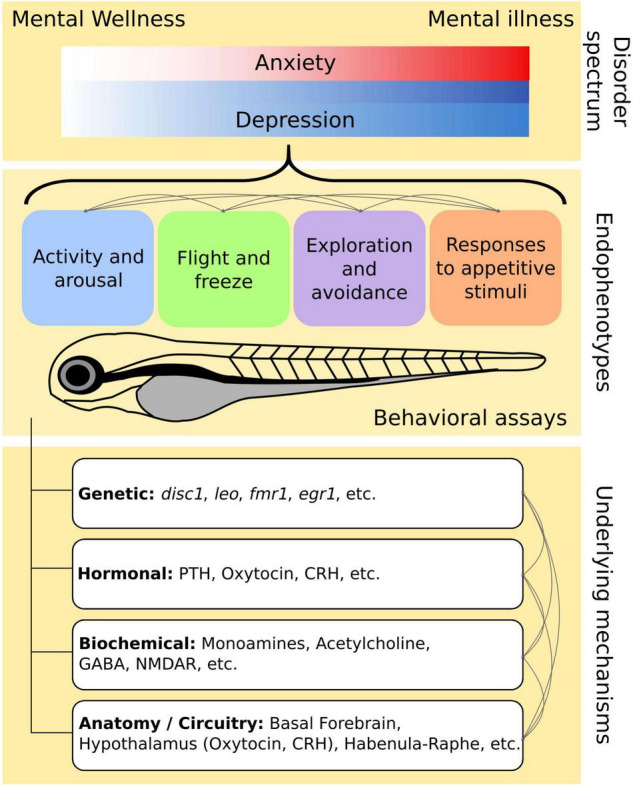
Conceptual relationship between endophenotypes and disease spectrum. Depression and anxiety exists along a spectrum of mental wellness to illness. There are overlapping and distinct aspects of these disorders that are also reflected in their shared and individual endophenotypes. Although we have categorized the endophenotypes into four main groups, these endophenotypes are interconnected and their relationships are worth investigating. The endophenotypes are supported by various underlying mechanisms, many of which are also interconnected. Multiple mechanisms may govern a single endophenotype while likewise, many endophenotypes can share similar pathways.

These mental illnesses have high prevalence in the population as described in Section “Population Mental Health and Endophenotypes.” In addition, they tend to exist as a continuum – patient diagnosis occurs for more severe cases where the disorder interferes significantly with their daily lives; however, many undiagnosed individuals span the spectrum between mental wellness and illness and could also benefit from measures to improve mental health (Figure 1).

Current treatments for mental illnesses are directed at symptom management. Thus, we dissect these 2 mental disorders into simpler behavioral markers (Table 2) by considering their symptoms and diagnosis criteria. We also classify the endophenotypes into four broad categories: (1) Activity and Arousal, (2) Flight or Freeze, (3) Exploration and Avoidance, and (4) Responses to Appetitive Stimuli (Figure 1).

**TABLE 2:** Summary of endophenotypes.

General category	Human endophenotype	Fish behavioral assay (endophenotype)	Specificity to mental health phenomena	Mechanisms (non-exhaustive)
Activity and arousal	Catatonia or restlessness	Hypoactivity or hyperactivity	Low	Multiple (De Marco et al., 2013, 2016; Irons et al., 2013; Woods et al., 2014; Vom Berg-Maurer et al., 2016; Barrios et al., 2020; Corradi et al., 2022)
	Sleep dysregulation	Sleep initiation/sleep maintenance (arousal threshold)/sleep	Medium	Multiple (Prober et al., 2006; Chiu et al., 2016; Rihel et al., 2010)
	Reduced arousal threshold	Light-dark transition	Medium	Monoamines, HPA (De Marco et al., 2013, 2016; Irons et al., 2013; Sveinsdóttir et al., 2022)
Flight or freeze	Freezing/immobility	Freezing	Medium	Left dorsal habenulo-interpeduncular pathway (Duboué et al., 2017)
	Increased startle responsiveness	Escape and startle potentiation/habituation	Medium	NMDAR, Pth, *fmr1*, serotonin (Roberts et al., 2011; Pantoja et al., 2016; Randlett et al., 2019; Anneser et al., 2022; Marquez-Legorreta et al., 2022)
	Heightened response to fearful stimuli	Larval alarm (Schreckstoff) response	High	Habenula-raphe, dopamine, serotonin (Basnakova et al., 2021; Jesuthasan et al., 2021)
	Learned helplessness	Active avoidance/passive coping	High	Habenula-raphe, glia-norepinephrine (Andalman et al., 2019; Lee et al., 2019; Mu et al., 2019)
	Other defensive behaviors	Nocifensive behavior/large-angle tail bends, aggression	Medium	Oxytocin, HPA (Wee et al., 2019a; Lovett-Barron et al., 2020)
Exploration and avoidance	Avoidance of risk/unfamiliar spaces	Light/dark avoidance	High	Serotonin, CRH (Steenbergen et al., 2011; Wagle et al., 2022)
	Avoidance of risk/open spaces	Thigmotaxis	Medium	GABA (Schnörr et al., 2012)
	Avoidance of risk/unfamilar spaces	Novel tank diving	High	Monoamines (Maximino et al., 2013b)
Responses to appetitive stimuli	Appetite dysregulation	Feeding assay	Low	Multiple (Jordi et al., 2018; Wee et al., 2019b)
	Stress-induced anorexia	Salt stress feeding assay	High	HPA (De Marco et al., 2014; Cheng et al., 2022)
	Avoidance of social eating	Social feeding assay	Medium	Oxytocin (Wee et al., 2022)
	Social avoidance	Social aggregation/avoidance/ orienting/preference assays	Medium	Pth, *disc1*, *egr1*, monoamines, basal forebrain (Tunbak et al., 2020; Harpaz et al., 2021; Anneser et al., 2022; Tallafuss et al., 2022)
	Response to addictive substances	Preference/self-administration assays	High	HPA, monoamines, glutamate, cholinergic system (Darland and Dowling, 2001; Ninkovic et al., 2006; Mathur and Guo, 2010; Bossé and Peterson, 2017; Müller et al., 2020)

### Activity and Arousal

Changes to activity and arousal levels are observed in mood and anxiety disorders. Agitation (e.g., fidgeting, pacing, and restlessness) and retardation (e.g., lethargy) represent two ends of the activity level spectrum but are often exhibited in mood and anxiety disorders (American Psychiatric Association, 2013). Individuals also tend to experience sleep disturbances, particularly insomnia, which is the result of lowered arousal thresholds (Riemann et al., 2010).

#### Locomotion

In mood and anxiety disorders, humans may display hypo- or hyperactivity. Hypoactive behaviors include reduced movement, staring, or selective mutism. In extreme cases, patients can develop catatonia, which if left untreated, has lethal effects (Wilcox and Reid Duffy, 2015). On the other hand, some patients with anxiety may be restless, easily distracted, and irritable. In severe cases, patients can be diagnosed with agitated depression (Park, 2018).

Locomotor behavior can be easily studied in zebrafish larvae. Various locomotion parameters such as swim speed, or detailed bout kinematics (interbout intervals and bout duration) can be obtained, both spontaneously and in response to stimuli, and may help distinguish increases or reductions in locomotion from specific defensive behaviors such as escapes and freezing (see Section “Flight or Freeze”) or sleep behavior (see Section “Sleep”) (Prober et al., 2006; Ingebretson and Masino, 2013). In zebrafish, environmental stressors such as low pH, high salt, high temperatures and water flow have been shown to cause changes in locomotion (Clark et al., 2011; De Marco et al., 2016; Vom Berg-Maurer et al., 2016; Lee et al., 2019; Cleal et al., 2020) which correlates also with the recruitment and increased synchronization of CRH neuron (part of HPA axis) activity (Vom Berg-Maurer et al., 2016). CRH neurons and downstream pituitary corticotrophs (De Marco et al., 2013, 2016) have been shown to modulate zebrafish locomotor responses to these, as well as other stimuli, however, the precise direction of locomotor change appears to be context-dependent (see Sections “Sleep,” “Light-Dark Transition,” “Freezing,” and “Other Defensive Behaviors” for elaboration).

Multiple neuropeptidergic pathways regulate locomotion in zebrafish, as studied in detail in Woods et al. (2014). In another study, galanin-producing neurons in the zebrafish preoptic hypothalamus were found to be activated by hyperosmotic stress. The ablation of these neurons resulted in significant stress-induced hyperactivity, suggesting the galanin-producing neurons are involved in the inhibition of the stress response. The HPA axis was also more active in larvae with ablated galanin-producing neurons, as indicated by higher cortisol levels (Corradi et al., 2022). In mammals, the neuropeptide galanin similarly regulates the stress response, particularly via the HPA axis to prevent overactivation which is often the case in stress-related anxiety and mood disorders (Karlsson and Holmes, 2006; Picciotto et al., 2010; Juhasz et al., 2014).

Another important regulator of zebrafish locomotor activity is the dopaminergic system. Dopamine receptor agonist apomorphine caused increased hyperactivity at low and high doses while antagonist butaclamol caused hypoactivity at high doses (Irons et al., 2013). Zebrafish have multiple dopaminergic populations that modulate locomotion (Barrios et al., 2020). Notably, dopamine plays a major role in anxiety (Zarrindast and Khakpai, 2015) and mood disorders (Belujon and Grace, 2017).

Hypoactivity or decreased locomotion could result in decreased exploratory behavior (see also Section “Exploration and Avoidance”) as the range of novel space that can be explored is reduced. In rodents (Crawley, 1985) and zebrafish (Stewart et al., 2012), decreased exploratory behavior is also frequently observed in anxiety models. Thus, the locomotion assay can be used to test the anxiety endophenotypes relating to both activity and exploration. However, locomotion can also be affected by a multiplex of causes such as muscle or nerve impairment, and can be influenced by many genetic, developmental or nutritional factors. Hence, it is not a specific endophenotype for anxiety and mood disorders and cannot be used exclusively.

#### Sleep

Sleep dysregulation is a common symptom in mood and anxiety disorders. Patients often face trouble maintaining sleep, resulting in early awakening, or difficulty initiating sleep, resulting in insomnia. Furthermore, sleep disturbances worsen these mental disorders, resulting in a positive feedback loop (Yang et al., 2022). Sleep-enhancing drugs are often prescribed in anxiety or mood disorders (Saletu-Zyhlarz et al., 2003; World Health Organization, 2009). Zebrafish exhibit similar sleep-like behavior primarily at night as regulated by their circadian cycle (Chiu and Prober, 2013). Like humans, zebrafish have characteristic sleep postures, increased arousal threshold during sleep as well as after sleep deprivation. Additionally, hypnotic or anesthetic drugs can be used to induce sleep and a sleep-like brain-state in both humans and zebrafish (Zhdanova et al., 2001; Leung et al., 2019). Regulation of sleep in humans and zebrafish is also conserved at the molecular level (Corradi and Filosa, 2021). The human Hypocretin Neuropeptide Precursor (HCRT) and zebrafish homolog hcrt decrease the arousal threshold when overexpressed, resulting in insomnia in humans and increased locomotion in zebrafish (Prober et al., 2006). Norepinephrine, an acute stress response hormone and neurotransmitter, has been shown to be a downstream effector of hcrt activity in zebrafish (Singh et al., 2015). Hypocretin is a neuropeptide involved directly in sleep regulation but a hyperactive hypocretin system is also linked to anxiety and depression. For instance, hypocretin activity results in wakefulness which is associated with heightened vigilance that is common in anxiety disorders (Johnson et al., 2012) while hypocretin protects against depression by promoting stress resilience (Nollet and Leman, 2013; Ji et al., 2019).

A rest/wake behavioral assay can be used to screen for drugs that modulate sleep (Prober et al., 2006; Rihel et al., 2010) in a high-throughput manner. Rihel et al. (2010) tested several classes of drugs that are used in anxiety and mood disorder treatment [e.g., serotonin, dopamine, GABA (Lydiard, 2003), epinephrine (Dooley, 2015) agonists and antagonists]. The drugs varied in their effects on different aspects of rest. For example, dopamine D2-receptor agonists reduced waking activity and increased rest while antagonists increased both waking activity and rest. Investigating drug effects on sleep might identify more specific mechanisms or treatments for mood or anxiety-induced sleep dysregulation.

The neuropeptide neuromedin U (Nmu) causes a reduction in sleep, and hyperactivity (Chiu et al., 2016). Overexpression of Nmu in larval zebrafish increased activity both in the day and at night, reduced sleep initiation and led to poorer sleep maintenance, suggesting a reduction in arousal threshold. A CRH receptor 1 (CRHR1) antagonist blocked the Nmu overexpression-induced waking hyperactivity in a dose-dependent manner. Hence, just as CRH is involved in locomotion regulation (see Section “Locomotion”), CRH also mediates the sleep and locomotion-related effects of Nmu overexpression.

There are certainly correlations between measures of sleep and locomotion (see also Cheng et al., 2022) though sleep can be distinguished from general activity using specific criteria (Prober et al., 2006). Similar to locomotion, sleep dysregulation itself is not exclusively an endophenotype for mental disorders, and needs to be considered alongside other more specific behaviors.

#### Light-Dark Transition

In the light-dark transition assay, zebrafish larvae are subjected to 5–30 min periods of whole-field darkness. During light periods, the larvae display low levels of activity while in the dark, they are hyperactive, which may reflect a heightened anxiety state (Irons et al., 2010; Golla et al., 2020). Chronically stressed (from 10 to 17 dpf) larval zebrafish displayed increased locomotion in response to the light-dark transition compared to non-chronically stressed counterparts (Golla et al., 2020). This light-dark transition assay was carried out in the light/dark partitioned well to simultaneously study light/dark choice (described in Section “Light/Dark Choice”) (Golla et al., 2020). However, the control and the group that had been exposed to chronic stress did not exhibit any difference in light/dark preference, suggesting a dissociation between these behaviors.

In another study, addition of anxiolytic compounds ethanol and cocaine caused a reduction in hyperactivity during dark periods while anxiogenic D-amphetamine caused an increase in hyperactivity during the dark period (Irons et al., 2010). Furthermore, increased thigmotaxis (see Section “Exploration and Avoidance”) was seen in dark periods compared to light periods even in control conditions. When anxiolytic aconitine was added, the extent of thigmotaxis behavior observed in the dark periods was reduced significantly (Ellis et al., 2012). In a recent study we observed that acute salt stress increased thigmotaxis in a light/dark choice assay (see Section “Light/Dark Choice”) relative to uniform light condition, suggesting a potential interaction of darkness with thigmotactic behavior (Cheng et al., 2022).

Monoaminergic systems have been implicated in both mood and anxiety disorders, and many antidepressants and anxiolytic drugs target monoamine function (Heninger and Charney, 1988; Tyrer and Shawcross, 1988; Delgado, 2000; Meyer et al., 2006; Liu et al., 2018). The vesicular monoamine transporter 2 (Vmat2) transports monoamines such as dopamine, serotonin, norepinephrine, and histamine into secretory vesicles for release from monoaminergic cells of the nervous system. *Vmat* mutants have reduced levels of the aforementioned monoamines as well as increased thigmotaxis (see also Section “Thigmotaxis”) (Sveinsdóttir et al., 2022; Wang, 2022). The *vmat* mutant larvae displayed a larger increase in movement immediately after dark transition but also reduced locomotion after the light was turned back on compared to wild-type larvae (Sveinsdóttir et al., 2022). However, neither dopamine agonist pramipexole nor precursor L-Dopa could rescue the immediate peak in activity after dark transition, suggesting the involvement of other monoamines or mechanisms (Sveinsdóttir et al., 2022).

While the above evidence supports the use of light-dark transitions as a measure of anxiety, there may be other mechanisms underlying dark hyperactivity that could confound the interpretation of results. For example, the hyperactivity observed in the dark could also be a sensorimotor response to environmental change. Heightened locomotion in the dark has been shown to be mediated by deep brain photoreceptors as a non-visually-mediated undirected photokinesis reflex (Fernandes et al., 2012). This reflex allows the larvae to swim to illuminated areas without having to visually detect light and orientate towards it like in phototaxis behavior. If and how this reflex relates to anxiety pathways remains to be seen. Given such alternative explanations, caution needs to be taken in interpreting this assay as a specific anxiety endophenotype.

Additionally, light of different wavelengths may also induce different behavioral responses. In a variation of the light-dark transition assay, De Marco et al. (2013, 2016) also showed that in dark-adapted larvae, blue or yellow light exposure induced hypoactivity during the period of exposure followed by recovery following light offset. Optically enhancing pituitary corticotroph activity via blue light stimulation of *Beggiatoa* photo-activated adenylyl cyclase (bPAC+) larvae further reduced locomotor activity, which also correlated with an increase in whole-body cortisol. Hence, blue/yellow light-induced hypoactivity can be potentiated by HPA axis activation downstream of CRH neuron activity. Further, under exposure to external stress (high temperatures and water flow), blue-light activated bPAC+ larvae exhibited enhanced locomotor activity, compared to controls in which CRH activation was solely through external stressor exposure. This means that pituitary corticotroph activity, which is downstream of CRH activity, potentiates and hence increases the magnitude of cortisol release and locomotor responses to stressors (De Marco et al., 2016). Such evidence points toward this assay as a promising anxiety endophenotype. Further, dark-adapted larvae avoided blue light and showed enhanced escape behaviors in a blue-light versus dark choice assay. Hence, HPA axis activation at multiple levels regulates diverse anxiety behaviors.

### Flight or Freeze

Across all species, predator, pain, and other aversive stimuli activate withdrawal or escape reflexes, or alternatively, a freezing response (Crawford et al., 1977; Blanchard et al., 1986; Blanchard, 1997; Roelofs, 2017). Anxiety and mood disorders have been shown to modulate these reflexes, as summarized in this section.

#### Freezing

Similar to mammals, freezing, or prolonged immobility, is a fear response in zebrafish and suggested to be the most reliable measure of anxiety in adults (Blaser et al., 2010). In larval zebrafish, freezing in response to an electric shock has also been observed, where freezing is defined as a period of inactivity exceeding 1.99s (Duboué et al., 2017). Severing the left fasciculus retroflexus (FR) caused an increase in freezing behavior post-shock while severing the right FR did not have a significant effect on shock recovery. The FR connects the dorsal habenula to the interpeduncular nucleus. These results suggest that the left dorsal habenulo-interpeduncular pathway is responsible for recovery from a heightened fear and anxiety state (Duboué et al., 2017). The HPA axis is also involved in the freezing response. A loss-of-function mutation to the glucocorticoid receptor ablated the negative feedback glucocorticoid cortisol provides to terminate the stress response (Gjerstad et al., 2018). This results in chronic HPA activation. Adult zebrafish with the mutation freeze for longer periods when placed in a novel tank and do not habituate even on subsequent exposures to a novel tank (Ziv et al., 2013). It remains to be seen if this freezing response in adults is related to stressor-induced hypoactivity observed in larvae (e.g., in response to water flow or blue light), since both are HPA-axis mediated (De Marco et al., 2016).

Since there are underlying neural circuits involved in freezing behavior, it can be an endophenotype for anxiety. Other genetic, molecular, and neural pathways may also interact to regulate freezing in zebrafish [see Sections “Alarm (Schreckstoff) Response” and “Learned Helplessness”].

#### Startle Responsiveness

Startle potentiation is a phenomenon where exposure to aversive stimuli causes an increase in the startle reflex. In humans, startle potentiation can be tested by measuring electromyographic activity of the orbicularis oculi muscle during blinking when shown aversive pictures. In humans, people with higher fear or anxiety levels display stronger startle, especially toward pictures relating to their phobias (Vaidyanathan et al., 2009). Repeated startle may lead to habituation where the stimuli no longer triggers a response; however, in some subjects, deficits in habituation may occur, which correlates with an anxiety-like state. Notably, Campbell et al. (2014) found a positive correlation between anxiety sensitivity and abnormal startle habituation.

Habituation and startle potentiation (Bhandiwad et al., 2018) has also been observed in zebrafish. Predator-like stimuli typically elicits escape reflexes such as O-bends and C-starts. O-bends enable reorientation of the body 180° to the direction of swimming and is commonly observed as a response to dark flashes (whole-field darkness) (Burgess and Granato, 2007). Vibrational-acoustic, mechanosensory and looming visual stimuli typically elicit C-starts mediated by the giant Mauthner cell and its homologs, during which the body curves in a ‘C’ shape to propel itself in a different direction from the stimulus (Hatta and Korn, 1998; Kohashi and Oda, 2008; Dunn et al., 2016). Randlett et al. (2019) developed a high-throughput dark-flash habituation assay where the aversive stimuli (darkflash) is delivered repetitively at spaced intervals. They found that the larvae gradually learnt not to respond to each darkflash, thus displaying habituation. The habituation response could be dissected into several components of behavior, and these were regulated by multiple molecular mechanisms (Randlett et al., 2019).

Multiple mechanisms of C-start habituation have also been discovered (Wolman et al., 2011; Corradi and Filosa, 2021; Nelson et al., 2022). For example, the persistent form of habituation response in zebrafish that lasts 25 min to 1 h could be inhibited by *N*-methyl-D-aspartate receptors (NMDAR) antagonists (Roberts et al., 2011). In humans, NMDAR antagonists such as ketamine have been used as antidepressants in mood (Amidfar et al., 2019) and anxiety disorders (Taylor et al., 2018b), though the precise mechanism behind the success of NMDAR antagonist action in humans still requires research (Sattar et al., 2018). In addition, deficiency of the neuropeptide parathyroid hormone 2 (Pth2) causes increased responsiveness in larval zebrafish to vibrational startle and reduces the delay between startle and response (Anneser et al., 2022). Larval zebrafish with reduced serotonin levels in the dorsal raphe nucleus (DRN) had increased habituation to acoustic startle, whereas optogenetic activation or a serotonin agonist was able to reduce habituation. This evidence points toward a significant role of DRN serotonin activity in startle habituation (Pantoja et al., 2016).

Fragile X syndrome arises from the silencing of the *fmr1* gene, causing habituation deficiencies in humans, mice, and zebrafish (Restivo et al., 2005; Kim et al., 2014; Ethridge et al., 2016; Marquez-Legorreta et al., 2022). *Fmr1* mutants displayed slower habituation to looming visual stimuli compared to wild type. Brain-wide imaging attributed this to overly-connected neural networks that failed to disconnect upon sensorimotor learning during habituation, leading to hypersensitivity in *fmr1* mutants (Marquez-Legorreta et al., 2022). Overall, changes in habituation kinematics could potentially be used as an endophenotype for anxiety, although they may also reflect more general differences in sensorimotor learning circuits.

#### Alarm (Schreckstoff) Response

Schreckstoff is an alarm substance that is released when a fish is injured. In adult zebrafish, this substance serves to alert the shoal to danger and therefore triggers a fear response indicated by darting and freezing behavior (Speedie and Gerlai, 2008; Mathuru et al., 2012; Chia et al., 2019). Alarm response behavior has recently been reported in larval zebrafish (Jesuthasan et al., 2021). After exposure to high concentrations of Schreckstoff, 50% of larval zebrafish [slightly lower than the 70% response rate observed in adults (Chia et al., 2019)] displayed a sustained increase in immobility of up to 20 min, which may reflect a prolonged heightened anxiety state. Consistently, neuroimaging showed a prolonged change in brain state after exposure to Schreckstoff in several areas (Jesuthasan et al., 2021). Of those areas the midbrain tegmentum, norepinephrinergic locus coeruleus and serotonergic raphe nucleus have been found to be involved in anxiety disorders (Lowry et al., 2008; Spiacci et al., 2012; DeGroot et al., 2020; Morris et al., 2020).

Interestingly, after exposure to Schreckstoff, larval zebrafish display reduced startle as compared to before (Basnakova et al., 2021). The authors’ interpretation of these results is that Schreckstoff exposure caused a heightened state of anxiety in the larval fish and hence increased their expectation of danger. The fish were therefore not startled when they encountered a second shock. Alternatively, the neural pathways regulating the Schreckstoff freezing response may override or inhibit those regulating startle. Disrupting *Clock* gene signaling in the habenula of larval zebrafish caused abnormal circadian regulation of dopamine and serotonin, and modulated Schreckstoff responses (Basnakova et al., 2021). Hence, both the acute Schreckstoff response and its influence on subsequent behaviors could potentially be useful endophenotypes for anxiety.

#### Learned Helplessness

Learned helplessness is the phenomenon where animals fail to exert control over aversive scenarios, which usually occurs after they are exposed to prolonged, unavoidable aversive events (Maier and Seligman, 2016). Depressed individuals display learned helplessness to a greater extent than healthy individuals. Miller et al. (1975) found that depressed subjects were more likely to wrongly perceive skilled responding as being up to chance. In other words, they believed they were helpless when in reality, they could learn to control the outcome. Hiroto (1974) experimentally demonstrated the learned helplessness effect in humans. During training, one group was able to control exposure to an aversive noise by pushing a button while for the other pressing the button had no effect. During the test, the group that had learned that they were helpless in dealing with the trauma did not attempt to end the trauma while the other two group took action. Hence, learned helplessness results in a reduced motivation to deal with difficult situations.

Animals will initially respond vigorously to an aversive stimulus but switch to a passive coping response when the inescapability of the stimulus becomes apparent (Koolhaas et al., 1999). In 10–15 dpf larval zebrafish, passive coping can be induced by inescapable shock and is characterized by immobility (Andalman et al., 2019). Whole-brain activity mapping revealed that the ventral habenula was uniquely hyperactive in the transition to passive coping. Modeling and optogenetic manipulation of neural connectivity also showed that aversive stimuli triggered significant changes in the intra-habenula and habenula-raphe connections, suggesting that the habenula-raphe pathway is involved in modulating the passive coping response (Andalman et al., 2019). In a prior study, 3–5 weeks old late larval/juvenile zebrafish were trained to avoid electric shock by moving away from a red light, a form of avoidance learning (Lee et al., 2010). When instead exposed to inescapable shock, the fish learnt that they were helpless and hence did not move away from the red light. The study also found that disabling the dorsal habenula using tetanus toxin caused larval zebrafish to behave as if they were exposed to inescapable shock even though they could have avoided the shock (Lee et al., 2010). Both the habenula and raphe nucleus have been implicated in human depression (Matthews and Harrison, 2012; Hahn et al., 2014; Gold and Kadriu, 2019). Hence, learned helplessness is a promising endophenotype for depression in larval zebrafish.

Larval zebrafish have also been shown to reduce their motivation to act in mildly aversive contexts. While they usually swim reflexively when encountering forward-flowing visual stimuli (an adaptive response that allows them to maintain their position in water currents) they will struggle and then become passive when the visual stimuli remains forward-flowing regardless of the fish’s swim attempts (Mu et al., 2019). Through activation and ablation experiments, the authors Mu et al. (2019) showed that glial cells play a critical role in integrating information from multiple failed attempts, which they receive from norepinephrinergic circuits, leading to the behavioral switch to passiveness. Hence, both neuronal and non-neuronal populations are involved in modulating the animal’s propensity to react to unsuccessful behavior. Given the distinct mechanisms, whether this phenomenon is related to learned helplessness or is enhanced in a depressive brain state remains to be seen.

#### Other Defensive Behaviors

Noxious stimuli activate the nociceptive system of animals, to trigger the conscious perception of pain or aversion (Pogatzki-Zahn and Schaible, 2020). Different noxious stimuli activate distinct pathways for pain perception in humans which have been reviewed extensively by Dubin and Patapoutian (2010). In zebrafish, hypothalamic oxytocin neurons transform noxious stimuli to motor output, evoking large-angle tail bends that can be described as a defensive response (Wee et al., 2019a). Lovett-Barron et al. (2020) similarly found that oxytocin neurons as well as other neuropeptidergic populations in the preoptic hypothalamus respond to homeostatic threats such as salinity, acidity, and heat. Calcium imaging, optogenetic and ablation experiments confirmed that both hypothalamic oxytocin and CRH neurons contribute toward these defensive behaviors via downstream brainstem targets which are distinct from those mediating escape responses (Wee et al., 2019a; Lovett-Barron et al., 2020).

Anomalies in sensitivity to noxious stimuli, magnitude of reaction, and the duration taken to recover are possible endophenotypes for anxiety disorders. Notably, oxytocin has also been implicated in anxiety and pain in mammals, including humans (Grillon et al., 2013; Poisbeau et al., 2018).

Aggression can also co-occur with anxiety and depression (Sánchez and Meier, 1997; van Praag, 1998; Neumann et al., 2010; Blain-Arcaro and Vaillancourt, 2017). However, since aggression only develops after the larval stage, aggression assays have mainly been designed for older zebrafish, as summarized by Way et al. (2015). In the mirror-biting assay, aggression is measured based on how much time a zebrafish spends ‘fighting’ with its mirror image or exhibiting aggressive postures (e.g., nose touching the mirror). Juvenile zebrafish (1 month old) exhibit similar responses to various aggression-reducing compounds including caffeine and sildenafil (Gutiérrez et al., 2020). Genetic inactivation of the histamine H3 receptor (Hrh3) in adult zebrafish caused reduced aggression and heightened anxiety, likely via altered serotonergic signaling in the telencephalon and hypothalamus (Reichmann et al., 2020). This suggests a link between aggression and anxiety, although more research is needed as aggression modulation pathways have yet to be fully characterized.

### Exploration and Avoidance

Avoidance and the reduction of exploratory behavior are typically observed in anxiety disorders, which will be elaborated on in the subsections below. However, these behaviors could also be modulated in depression and other mood disorders (Cella et al., 2010; Blanco et al., 2013; Haskell et al., 2020). We will focus on light/dark choice, thigmotaxis and novel tank diving, however other assays for exploration and avoidance have also been developed, as discussed in Corradi and Filosa (2021).

#### Light/Dark Choice

The state of anxiety often triggers an increase in avoidance when presented with an approach-avoidance conflict (AAC). An AAC occurs when two situations – one safe and one risky – are present simultaneously. A heightened anxiety state would typically cause the subject to choose the safer options over riskier unknowns (Aupperle et al., 2011). In rodent models, a choice between light and dark presents an AAC where the dark zone is perceived as safer. Imaizumi and Onodera (1993) found that the addition of anxiogenic drugs zolantidine and thioperamide increased the time spent in the dark zone. In mice, the type 2 CRF receptor neurons in the lateral septum regulate anxiety by projecting to the anterior hypothalamus. Optogenetic studies to activate these neurons had an anxiogenic effect, where mice preferred to remain in the dark side of the light/dark partitioned box (Anthony et al., 2014). These studies suggest that behavior in a light/dark test could be an indicator of anxiety.

The light/dark choice assay has also been successfully implemented in zebrafish. Larval zebrafish exhibit a natural preference for light (phototaxis) and avoidance of dark (scotophobia). Notably a behavioral switch to dark preference has been reported in adulthood (Lau et al., 2011). Larval zebrafish are placed individually in an arena with a well-lit and dark region and the proportion of time in the light area can be used to quantify anxiety (Steenbergen et al., 2011). When the larvae are exposed to stressful stimuli such as UV light, their cortisol levels and dark-avoidance behavior increase (Bai et al., 2016). This provides evidence that increased dark avoidance is an indicator of heightened stress and anxiety levels. Hence, the light/dark choice assay could be used to assess anxiety state in zebrafish. However, it is worth noting that heat stress and mechanical disturbance did not trigger the expected correlated increase in dark avoidance and cortisol levels. We show that prolonged, but not acute osmotic stress enhances dark avoidance in larvae (Cheng et al., 2022). This could suggest that different stressors and stressor durations generate distinct anxiety states that are regulated by diverse pathways.

Steenbergen et al. (2011) further validated the larval zebrafish light/dark choice assay using clinically-established anxiolytic or anxiogenic compounds. They found that larval zebrafish spent more time in the dark region when treated with buspirone, a serotonin receptor (5-HT1A) agonist, while the converse was true for anxiogenic compounds such as caffeine. Hence, serotonin signaling as well as other conserved signaling pathways may share common roles in modulating anxiety across species. The role of serotonin in regulating zebrafish anxiety has been further supported by studies in adult zebrafish using novel tank, light/dark, light/dark plus maze assays (Sackerman et al., 2010; Maximino et al., 2013a; Nowicki et al., 2014).

Recently, hypothalamic CRH neurons were shown to promote dark avoidance behavior (Wagle et al., 2022). These CRH neurons displayed lower overall activity in the presence of light, and their ablation increased brainwide representations of light and reduced dark avoidance (Wagle et al., 2022). CRH receptor antagonism or *crhb* knockout similarly reduced dark avoidance. In contrast, oxytocin neuron ablation enhanced dark avoidance, suggesting distinct roles of hypothalamic peptidergic neurons in light/dark choice regulation.

Additionally, dark avoidance is a form of reduction in exploratory behavior as the animals show reduced propensity to enter unfamiliar places. Such decreased exploratory behavior has been observed in anxiety models in both rodents (Crawley, 1985) and zebrafish (Stewart et al., 2012). Overall, the light/dark choice assay might be one of the more specific anxiety endophenotypes in zebrafish.

#### Thigmotaxis

Thigmotaxis, or wall-hugging behavior, is also conserved in many animal species. The fear of exposure to predators in open spaces causes animals to prefer staying close to the perimeter of the environment. Thigmotaxis can also be interpreted as a reduction in exploratory behavior as there is a reduction in willingness to enter the unfamiliar open space. Decreased exploratory behavior has been observed in animal models of anxiety (Crawley, 1985; Stewart et al., 2012). An open field test showed that humans with high anxiety sensitivity and agoraphobia also have a higher tendency to keep to the edges of the arena while their healthy counterparts spent most time in the center of the arena (Walz et al., 2016).

Similarly, larval zebrafish display increased thigmotaxis in stressed conditions such as in the presence of chemical irritants (Roberts et al., 2020). Therefore, a version of the open field test can be used as a measure of anxiety levels. Zebrafish larvae are placed in an arena and their movement is recorded for a set amount of time. Thigmotaxis is quantified as a percentage of the distance moved in the outer zone of the well out of the total distance moved. Anxiolytic drugs have been found to reduce thigmotaxis while anxiogenic compounds increase thigmotaxis. For example, Schnörr et al. (2012) found that diazepam, an FDA-approved drug for anxiety treatment, significantly reduced thigmotaxis. In humans, diazepam binds allosterically to gamma-aminobutyric acid (GABA) receptors in the limbic system, reducing the excitability of neurons, which mediates its anxiolytic effect. While the mechanism of diazepam action in zebrafish has not been precisely elucidated, diazepam has been shown to bind to GABA receptors in other species of fish (Wilkinson et al., 1983; Friedl et al., 1988).

Through selective breeding, Wagle et al. (2017) found that dark aversion and thigmotaxis are heritable traits. Fish that display strong dark avoidance also displayed increased thigmotaxis in an open field test, indicating increased propensity of being in a high anxiety state. This suggests that both thigmotaxis and dark avoidance have a genetic underpinning, making them candidate endophenotypes for anxiety.

In a recent study, we showed that acute salt stress enhances thigmotaxis in a light/dark choice assay, with thigmotaxis only during the light/dark period as compared to during uniform light. However, dark avoidance was unaffected, suggesting that dark avoidance and thigmotaxis behaviors are dissociable traits with potentially different underlying pathways. In contrast, prolonged, high salt stress enhanced dark avoidance but had weaker effects on thigmotaxis (Cheng et al., 2022).

However, thigmotaxis can also be induced by other biological phenomena such as seizures (Baraban et al., 2005), and may be influenced by experimental factors (see Section “Controls and Considerations in Endophenotype Investigation”), hence it should be interpreted with caution as an anxiety phenotype.

#### Novel Tank Diving

In the novel tank diving (NTD) assay, the zebrafish is placed into a narrow tank to constraint most of its movement to the vertical axis. The unfamiliar environment increases stress and anxiety, causing the zebrafish to initially dwell at the bottom of the tank. In the next 5 min, they gradually begin to explore the upper regions of the tank. NTD assays are typically conducted on adult zebrafish, but have been successfully conducted using 25 dpf larval zebrafish (Golla et al., 2020). Larval zebrafish exposed to chronic stress (net-chasing, water turbulence, salt stress, low pH, and light flashes applied randomly during the day) have increased bottom-dwelling times (Golla et al., 2020) which is in agreement with increased responsiveness to light-dark transition. However, they do not display increased thigmotaxis, dark avoidance and shoaling.

A variation of the NTD has been proposed for younger larvae. Seven dpf larvae were shown to display depth preference in the first 30 min of introduction to a novel tank, with caffeine enhancing, and diazepam alleviating bottom-dwelling behavior (Fontana and Parker, 2022).

In adults, the anxiolytics nicotine, diazepam and buspirone caused a reduction in the time spent bottom dwelling (Bencan and Levin, 2008; Bencan et al., 2009), suggesting that bottom-dwelling behavior is indicative of anxiety. The *leo* mutant strain has increased monoamine oxidase (MAO) activity and serotonin turnover rate, leading to increased thigmotaxis and light avoidance in adult zebrafish (Maximino et al., 2013b). In a NTD assay, *leo* mutant zebrafish displayed increased bottom-dwelling (Cachat et al., 2011; Maximino et al., 2013b), suggesting that there are genetic underpinnings to the NTD.

In addition to the propensity to explore novel environments, the NTD has been used to measure habituation in adult zebrafish (Wong et al., 2010). In an unfamiliar environment, time is needed for the zebrafish to acclimatize before normal behavior is resumed. As described above (see Section “Startle Responsiveness”), habituation deficits are also characteristics of anxiety-related disorders. Hence, the novel tank diving assay could be one of the more specific anxiety endophenotypes in zebrafish.

### Responses to Appetitive Stimuli

Disruptions in reward pathways of the brain have been associated with mood and anxiety disorders (Ulrich-Lai et al., 2010; Russo and Nestler, 2013). Atypical responses to pleasurable stimuli such as food or positive social interaction might be an indication of dysfunctional reward signaling which could be a sign of mood or anxiety disorders, as summarized in this section.

#### Appetite Dysregulation

Appetite, or the drive to consume food, is often dysregulated in mood and anxiety disorders, as observed behaviorally and supported by numerous studies (Rudenga et al., 2013; Weidenfeld and Ovadia, 2017). Feeding when stressed is correlated with reduced activation in reward areas of the brain (amygdala, hippocampus, and cingulate cortex), suggesting that stress causes reduced sensitivity to the pleasurable rewards derived from food, which leads to excessive eating (Born et al., 2010). Furthermore, excessive cortisol causes strong increases in appetite. This effect has been confirmed in studies on Cushing’s syndrome patients who face prolonged exposure to glucocorticoids, resulting in significant weight gain (Fleseriu et al., 2012), as well as depression and anxiety as comorbidities (Sonino and Fava, 2001). On the other extreme, stress-induced anorexia is also commonly observed in humans and other mammals [see Section “Stress Feeding Behavior/Stress-Induced Anorexia” (Shimizu et al., 1989; Liu et al., 2007; Guarda et al., 2015)]. Hence, appetite dysregulation can be an endophenotype for mood and anxiety disorders. However, since appetite is regulated by many homeostatic and hedonic factors, it is also not a specific endophenotype.

Zebrafish larvae begin feeding by 5 dpf, often hunting small aquatic organisms such as paramecia or rotifers. While paramecia hunting assays have been commonly used to quantify zebrafish feeding, they are relatively low-throughput and require specialized imaging setups (Gahtan, 2005; McElligott and O’malley, 2005). More recently, variations of gut fluorescence feeding assays (Shimada et al., 2012; Jordi et al., 2015, 2018; Wee et al., 2019b; Cheng et al., 2022) have been used to quantify paramecia intake in individuals or groups of larval zebrafish. Larvae are starved before fluorescently-labeled paramecia are added to the arena. Gut fluorescence can be imaged continuously using customized setups (Jordi et al., 2015, 2018), or alternatively, larvae can be anesthetized or fixed after specific time windows (e.g., 15 min) and fluorescently imaged (Wee et al., 2019b,^2021^; Cheng et al., 2022). This feeding assay can be adapted accordingly to study more specific aspects of appetite dysregulation, such as under stress or in social contexts, as described in the next sections.

#### Stress Feeding Behavior/Stress-Induced Anorexia

During stress, HPA activation causes increased release of cortisol, which has been shown to stimulate hunger and increased food intake (Tataranni et al., 1996). However, there is some evidence that the type of stress (e.g., physical vs. social, traumatic vs. non-traumatic) can have different effects on appetite (Adam and Epel, 2007). While some may binge when stressed, others might have reduced food intake. In rats, restraint and forced swimming stress results in anorexic behavior (Calvez et al., 2011). Regardless of the directional change in appetite, dysregulation of eating patterns is implicated in anxiety and stress-related disorders. Moreover, there is a high comorbidity between anxiety and eating disorders (Swinbourne et al., 2012), further supporting the relationship between dysregulated appetite and anxiety.

Larval zebrafish are freshwater fish, hence high salinity can be used as a stressor. De Marco et al. (2014) found that salt stress at 50 and 100 mM NaCl suppressed feeding motivation – in normal conditions, larval zebrafish would preferentially occupy the region of the arena that contained paramecia, whereas when placed in hyperosmotic salt solutions, the larvae occupied all regions equally. Since whole body cortisol levels of the larvae increased with increasing salt concentration, the HPA axis was activated by the salt stress (De Marco et al., 2014). To improve scalability, we have adapted our gut fluorescence feeding assay to examine stress-induced anorexia (Wee et al., 2019b; Cheng et al., 2022). Using this assay and a new machine-based gut segmentation approach, we found that both acute and prolonged salt exposure reduced paramecia consumption in a dose-dependent manner (Cheng et al., 2022). Given the high homology between the zebrafish and human HPA stress axes, and the fact that appetite dysregulation has been linked to this stress pathway, stress feeding behavior could be a viable endophenotype for anxiety disorders.

When stressed, humans tend to choose caloric dense food such as sweet, high-carbohydrate and high-fat snacks (Oliver et al., 2000; Zellner et al., 2006; Adam and Epel, 2007), often leading to stress-associated weight gain. The increase in consumption of such foods also leads to reprogramming of stress pathways and behavioral changes, as detailed by Hardaway et al. (2015). For example, carbohydrate-rich food is thought to increase serotonergic activity, thus decreasing anxiety and depression (Rogers, 1995). Consumption of these different meal compositions also likely has corresponding effects on their mood and stress levels (Born et al., 2010). It remains to be seen if these phenomena can be modeled in larval zebrafish.

#### Social Feeding Behavior

Social facilitation of eating is the phenomenon where individuals eat more when in groups than when alone (Zajonc, 1965; Higgs and Thomas, 2016; Herman, 2017; Ruddock et al., 2019). Similar observations have been made in many animal models (Harlow, 1932; Tolman, 1964; Reynaud et al., 2015), including freshwater fish (Uematsu and Ogawa, 1975). While it may not be possible to fully model such a complex phenomenon in zebrafish, the ability to regulate feeding based on social context could be a useful indicator of mental function, with abnormal reactions to social context (e.g., reduced feeding in groups) reflecting mental ill-being (Patel and Schlundt, 2001; Brown et al., 2003).

Larval zebrafish have been shown to eat more when in groups than alone (Wee et al., 2022). Larvae at 6–8 dpf were plated either individually or in small groups for 2 h and the amount of food consumed by each zebrafish was compared. Fish density and crowding were controlled for by limiting the groups to small numbers (e.g., 3 fish) in the same-sized arena as isolated fish (see also Section “Controls and Considerations in Endophenotype Investigation”). The authors found that acutely (2 h) isolated larvae displayed reduced feeding compared to the larvae that were in groups. Notably, the odor of kin conspecifics was sufficient to enhance feeding in isolated fish. The authors identified a olfactory-subpallial-oxytocinergic pathway that integrates conspecific social cues, and showed that ablation of the oxytocinergic neurons or application of oxytocin antagonists could rescue the effects of isolation on feeding (Wee et al., 2022). This oxytocinergic circuit also promotes defensive behavior (see Section “Other Defensive Behaviors”), which is similarly modulated by conspecific social cues (Wee et al., 2022). In humans, oxytocin neurons can be activated by stressful stimuli, and have been implicated in appetite regulation, aversive responses, and social behavior (Striepens et al., 2012; Lieberwirth and Wang, 2014; Neumann and Slattery, 2016). Further, the basal forebrain (subpallial) neurons connecting olfactory to oxytocin circuits have been previously implicated in visually-mediated social orienting behaviors, and may be conserved in mammals (Stednitz et al., 2018).

Alternatively, this social feeding assay can also be used to study isolation-induced anxiety. To socially-reared larval zebrafish, isolation activates overlapping brain regions as aversive or stressful stimulus, including the oxytocin network (Wee et al., 2022), hence the isolation state may also reflect an anxiety state that affects feeding (also see Section “Controls and Considerations in Endophenotype Investigation”). Currently, the effects of chronic social isolation on zebrafish feeding are still unknown – in Drosophila, this leads to enhanced, rather than reduced food intake as well as reduced sleep (Li et al., 2021). In short, by comparing the relative effects of interventions on isolated and group feeding behavior, it may be possible to quantify both anxiety and social behavior endophenotypes.

#### Social Preference or Avoidance Behavior

Social avoidance is a common theme in mood and anxiety disorders – patients become more isolated and partake in fewer social activities (Coyle and Dugan, 2012; Holt-Lunstad et al., 2015; Taylor et al., 2018a; Santini et al., 2020). Zebrafish, like humans, are a social species. Group behavior allows better defense against predators, more efficient hunting and improved foraging. In larvae, aggregation can be used as a proxy for social cohesion. Interestingly, younger larvae (7 dpf) have a natural tendency for mutual repulsion while older larvae (21 dpf) exhibit mutual attraction. Harpaz et al. (2021) suggests that this could be an adaptation to the lower oxygen levels in sheltered waters where younger larvae dwell. In the larval group assay, groups of 5 larvae were placed in custom arenas and aggregation was measured as the negative log of the sum of nearest-neighbor distance between each of the fish of test groups divided by that of the shuffled control groups. *Disc1* mutants, previously shown to have abnormal stress responses (Eachus et al., 2017), displayed increased social cohesion in this group assay (Harpaz et al., 2021). Interestingly, mutated forms of the human homolog DISC1 protein have been found in the brains of major depression patients (Sawamura et al., 2005). Overall there is potential for the group assay to be used to study social aggregation or avoidance in the context of anxiety and mood disorders.

Social avoidance in larval zebrafish is characterized by increased frequencies of high acceleration escapes and short latency c-start swim bout types upon social interaction. Mechanosensory stimuli in the form of water vibrations generated by a piezoelectric bender actuator mimics social interaction. When these water vibrations are detected within certain proximity, the larvae will attempt to swim to avoid what they perceive to be other fish. Larvae raised in isolation will react when vibrations are detected at closer proximity (smaller avoidance distance) compared to those raised in groups. Ablation of the lateral line, through which larval zebrafish sense water motion, caused larval zebrafish raised in isolation to have similar avoidance distance as those raised in groups (Groneberg et al., 2020). In addition, mechanosensory stimuli generated by the movements of nearby fish modulates the expression of *pth2*. Isolation of socially-reared zebrafish larvae caused a reduction in *pth2* transcripts (Anneser et al., 2020). *Pth* mutants not only show increased startle responsiveness (see Section “Startle Responsiveness”) but also reduced social preference and shoaling at late juvenile stages (Anneser et al., 2022).

The early growth response factor 1 (Egr1) has been implicated in depression in humans (Covington et al., 2010) and social anxiety-like behaviors in mice (Stack et al., 2010). In zebrafish, isolation induced downregulation of the *egr1* transcript (Anneser et al., 2020). In a different social interaction assay - the larval dyad assay (Stednitz and Washbourne, 2020) – the loss-of-function *egr1* mutant 14 dpf zebrafish spent less time near the divider between itself and another similar-size zebrafish (Tallafuss et al., 2022). Furthermore, it displayed less social orienting behavior, indicating weak social interaction behavior. In a biological motion assay (Larsch and Baier, 2018), the *egr1* mutants displayed less inclination to follow projected dots that had size and movement kinematics mimicking that of a 14 dpf larva (Tallafuss et al., 2022), also supporting the hypothesis that Egr1 is essential for normal social behavior. These *egr1* mutants also had fewer tyrosine hydroxylase 2 (Th2) expressing neurons in the basal forebrain. Ablation of 30% of this neuronal population in wild types yielded similar behavior as seen in the *egr1* mutants. Notably, the zebrafish basal forebrain contains other cell types, such as cholinergic neurons, which have been shown to be important for social development and function, and may interact with these Th2 neurons (Stednitz et al., 2018; Wee et al., 2022).

Another variation of the social preference assay allows larvae to choose between a region where it can see conspecifics and a region where it cannot. The assay is conducted using 14–21 dpf larval zebrafish in which social preference has already developed (Dreosti et al., 2015). Larvae raised in groups displayed social interaction behavior, orienting themselves at a 45-degree angle and synchronizing their motion with conspecifics. Conversely, larvae raised in isolation were found to freeze or simply watch the conspecifics. Treatment of isolated larvae with buspirone (a 5-HT1A receptor agonist) rescued such anti-social behavior, further supporting an involvement of monoamines in the regulation of social behavior (Tunbak et al., 2020).

Overall, social avoidance or abnormal social interactions have been shown to be supported by multiple underlying circuit and hormonal mechanisms and is a candidate mood and anxiety endophenotype in larval zebrafish.

#### Responses to Addictive Substances

There is a high comorbidity between drug/alcohol addiction and mood or anxiety disorders (Robbins, 1974; Merikangas et al., 1998; Grant et al., 2004). The self-medication hypothesis suggests that individuals first develop mood or anxiety disorders as a result of external stressors or trauma before they turn to drug abuse to cope through a form of ‘self-medication’ (Khantzian, 1985). Over time, this develops into an addiction. Several assays have been developed for mainly late larval/juvenile (> 3–4 weeks) and adult zebrafish to study addiction behaviors, including conditioned place preference (CPP) assays where the animal associates the drug with a neutral environmental stimulus (Tzschentke, 2007; Webb et al., 2009; Mathur et al., 2011; Collier and Echevarria, 2013) and self-administration assays in which the zebrafish triggers the delivery of addictive substances by its own actions (Bossé and Peterson, 2017; Nathan et al., 2022). Müller et al. (2020) reviews these and additional assays in more detail. Addictive drugs such as nicotine, ethanol, opioids, and cannabinoids have also been shown to affect anxiety and endophenotypes in both zebrafish and mammals, such as light/dark choice and light-dark transition (Irons et al., 2010; Steenbergen et al., 2011). Conserved genes and pathways such as the dopaminergic and cholinergic systems have been shown to underlie addictive drug responses (Darland and Dowling, 2001; Ninkovic et al., 2006; Mathur and Guo, 2010). As there are many shared mechanisms between addiction behaviors and mood and anxiety disorders (Goeders, 2004; Lüthi and Lüscher, 2014), behavioral responses to addictive substances could be useful endophenotypes particularly for cases of addiction and mood/anxiety disorder comorbidity.

## Controls and Considerations in Endophenotype Investigation

As described in Section “Human and Zebrafish Anxiety and Mood Endophenotypes,” multiple assays can be used to characterize endophenotypes in different mental disorders using the larval zebrafish model. For many assays, variations in specific protocols could potentially affect interpretation of results. It is therefore important to consider the choice of protocol in relation to the aims of the study. Here, beyond protocol differences, we briefly describe other variables that can affect experimental outcomes, which may need to be controlled or accounted for Table 3.

**TABLE 3:** Summary of factors influencing behavioral assay outcomes.

Factor	Description/assays affected (non-exhaustive)	References
Age	Learned helplessness, locomotion, light/dark choice and transition, social aggregation/preference/orienting	Lee et al., 2010; Lau et al., 2011; Padilla et al., 2011; Valente et al., 2012; Dreosti et al., 2015; Andalman et al., 2019; Stednitz and Washbourne, 2020
Arena size and depth	Locomotion	Padilla et al., 2011; Ingebretson and Masino, 2013; Christou et al., 2020
Genotype/strain	Light-dark transition, startle habituation, stress-induced anorexia	O’Malley et al., 2004; van den Bos et al., 2017; Cheng et al., 2022
Feeding state and diet	Locomotion, social preference/orienting, thigmotaxis, feeding	Clift et al., 2014; Filosa et al., 2016, Wee et al., 2019b; Stednitz and Washbourne, 2020
Environmental state	Feeding, locomotion, light/dark choice, sleep, startle habituation	McHenry et al., 2009; Clark et al., 2011; Olszewski et al., 2012; Suli et al., 2012; Yeh et al., 2013; De Marco et al., 2014, 2016; Bai et al., 2016; Olive et al., 2016; Oteiza et al., 2017; Bhandiwad et al., 2018; Cheng et al., 2022
Crowding/fish density	Thigmotaxis, locomotion, social aggregation/avoidance, feeding, defensive behavior, startle responsiveness	Burgess and Granato, 2008; Zellner et al., 2011; Delomas and Dabrowski, 2019; Groneberg et al., 2020; Zaki et al., 2021; Wee et al., 2022
Time of day	Locomotion and arousal, startle responsiveness	Zhdanova et al., 2001; Hurd and Cahill, 2002; Prober et al., 2006; MacPhail et al., 2009; Randlett et al., 2019; Basnakova et al., 2021
Microbiome	Locomotion, thigmotaxis, social orienting	Davis et al., 2016; Phelps et al., 2017; Bruckner et al., 2020; Weitekamp et al., 2021

### Age

While our review focuses on larval stages, many studies have shown a strong age dependence of behaviors even within the first 4 weeks of development. For example, in an operant conditioning task, Valente et al. (2012) found that the ability to learn began at week 3 and reached the max performance index at week 6. Hence, many learned helplessness and addiction assays are conducted at later ages (Lee et al., 2010; Andalman et al., 2019; Müller et al., 2020). Similarly, larval age was found to be a factor affecting locomotion in light-dark transitions (Padilla et al., 2011). In light periods, larval zebrafish increased their locomotor activity as they developed from 4–6 dpf (Padilla et al., 2011). Age was similarly shown to affect baseline locomotion and locomotor variability in a different study (Ingebretson and Masino, 2013). Larval zebrafish are known to exhibit scotophobia in the light/dark choice assay but Lau et al. (2011) has shown that the maturation of fishes into adulthood reversed this paradigm and resulted in light avoidance behavior. Finally, age also influences social behavior. Multiple studies have shown that preference for conspecifics and social aggregation or orienting tends to occur only after 2 weeks of age (Dreosti et al., 2015; Stednitz and Washbourne, 2020; Harpaz et al., 2021), although other social behaviors emerge sooner (Wee et al., 2022).

### Arena Size and Depth

Media volume used in arenas was also found to affect larval zebrafish behavior. Christou et al. (2020) reported that the distance moved and activity time of 4–7 dpf larval zebrafish increased when more media volume was used in wells. Similarly, locomotor activity was found to be affected by well depth in an age-dependent manner (Ingebretson and Masino, 2013). While Ingebretson and Masino (2013) showed that variability but not motility was affected by wells of different diameters, Padilla et al. (2011) demonstrated an effect of arena size on larvae locomotion, with larvae kept in 24-well plates exhibiting more movement than larvae kept in 48 and 96-well plates. Other behaviors such as thigmotaxis are also likely affected by arena size (Cheng et al., 2022).

### Genotype/Strain

The use of different wild-type strains, such as the AB and Tüpfel long-fin (TL), can also produce variable results. In the light-dark transition assay, both strains responded differently to light-dark/dark-light transitions, with AB strains showing stronger behavioral responses (van den Bos et al., 2017). Furthermore, AB strains have been demonstrated to display stronger habituation to repeated acoustic stimuli and thus showed weaker responses when compared to TL strains (van den Bos et al., 2017). The use of nacre mutants in imaging protocols has been favored due to their low pigmentation and transparency. Differences in behavior between nacre and wild-type fishes have not been fully studied, but O’Malley et al. (2004) has shown that nacre larvae displayed locomotor behaviors identical to those of wild-type larvae. We also found that salt stress-induced anorexia differed across AB, TL and nacre genotypes, with AB fish showing the strongest reduction in feeding (Cheng et al., 2022).

### Feeding State and Diet

The effects of feeding on different behavioral parameters have also been studied. Relative to unfed fish, fed 6 dpf larvae displayed differences in avoidance responses, swim speeds and resting periods, while fed 7 dpf larvae additionally displayed changes in thigmotaxis and social distancing behaviors (Clift et al., 2014). These changes in behaviors might be associated with increased energy availability in the fed state, but did not imply that the behaviors were linked (Clift et al., 2014). When fed with dry food, larvae were found to have shorter body lengths and displayed social deficits, suggesting that diet is an important factor in social behavior (Stednitz and Washbourne, 2020). Hunger state also influences feeding-related endophenotypes and threat avoidance behaviors. Filosa et al. (2016) demonstrated an effect of feeding status on the propensity to avoid an ambiguous visual stimulus representing either prey or predator, which is mediated by serotonergic raphe neurons and the HPA axis. Wee et al. (2019a,b) found that neuronal populations such as the caudal hypothalamus are convergently activated by starvation and aversive stimuli. Hence, it is possible that the feeding state can affect anxiety/depression-like behaviors via convergent neural pathways.

### Environmental State

Not surprisingly, environmental context, such as water quality, temperature, noise, or water flow/agitation could similarly affect the manifestation of anxiety or depression-like behaviors, and should be avoided or controlled for in behavioral assays. As described above, a hyperosmotic environment and mechanosensory stress can increase cortisol and reduce feeding, and can themselves be utilized as a behavioral assay for stress-induced anorexia (De Marco et al., 2014; Cheng et al., 2022). Clark et al. (2011) found that hyperosmotic shock caused a dose-dependent effect on larval fish movement. However, De Marco et al. (2014) reported no changes in locomotor activity due to salt stress. Our experiments suggest that prolonged osmotic stress reduces sleep while leaving swim speed mostly intact (Cheng et al., 2022). Temperature changes (cold or hot stressors) have also been shown to affect locomotion and dark avoidance behaviors (Bai et al., 2016). Changes in pH also increased cortisol levels in fish, with cortisol levels being correlated to HCl concentration (Yeh et al., 2013).

Environmental noise levels can alter behavioral and physiological responses of larvae. Prolonged exposure to 20 dB re 1 ms^–2^ flat-spectrum noise caused decreased startle response thresholds and increased sensitization to startle stimuli (Bhandiwad et al., 2018). These noise-exposed larvae also displayed reduced locomotor activity (Bhandiwad et al., 2018). Water flow stimuli can also evoke an escape, avoidance, or hypoactive locomotor response in larvae, correlating with cortisol levels (McHenry et al., 2009; Olszewski et al., 2012; De Marco et al., 2016), and continuous water flow also induces rheotaxis (Olszewski et al., 2012; Suli et al., 2012; Olive et al., 2016; Oteiza et al., 2017). Hence, excessive agitation should be avoided before and during behavioral experiments.

### Crowding/Fish Density

Fish density has also been found to affect zebrafish behavior. As described in Section “Other Defensive Behaviors” and “Social Feeding Behavior,” the presence or absence of conspecifics influences feeding and defensive behavior (Wee et al., 2022). Circling behavior – defined as multiple conspecifics moving in an organized manner along the edges of the arena towards one direction – was observed in dishes with dense zebrafish populations and occurred more frequently along with increased densities (Zaki et al., 2021). Furthermore, it was also shown that larvae in dense arenas preferred edges and the outer circumferences of the arena. These behaviors resemble thigmotaxis as observed in anxious zebrafish, though it is unclear if it is an anxiety phenotype. Isolation also affects larval behavioral responses. Singly-reared larvae were found to exhibit lower locomotor activity in dark periods compared to group-reared larvae (Zellner et al., 2011). Larvae raised in isolation had impaired social preference (Tunbak et al., 2020), increased social avoidance, and also swam fewer but longer bouts (Groneberg et al., 2020). Burgess and Granato (2008) reported that larvae raised in lower density had increased sensitivity to startle. Aggregation behavior is also likely affected by fish density. Similarly, feeding increases with fish density though overcrowding may counteract this effect (Wee et al., 2022). Crowding stress was affected by tank size and water volume in adult zebrafish, but studies on crowding stress have not been reported in larval zebrafish. However, holding densities of larvae were also inversely related to larval growth, with higher densities (>22 fish/L) leading to lower mean individual larvae weights (Delomas and Dabrowski, 2019). Thus, adhering to a standard raising protocol for zebrafish larvae is important to avoid complications from confounding factors.

### Time of Day

MacPhail et al. (2009) tested the locomotion of larvae from 10:00 to 15:30 h in darkness using infrared and found that locomotor behavior was affected by time of day. Activity was highest when testing began and decreased to a stable level by the afternoon, with no changes being noted between 13:00 to 15:30 h (MacPhail et al., 2009). At night, larvae display lower activity and characteristics of sleep, including increased arousal thresholds (Zhdanova et al., 2001; Hurd and Cahill, 2002; Prober et al., 2006). Disturbances to sleep also affected locomotion. Mechanical stimuli applied during the last 6 h of night resulted in decreased daytime locomotor activity and increased arousal thresholds in the following day (Zhdanova et al., 2001). Larvae display stronger dark flash habituation responses in subjective night versus day phases (Randlett et al., 2019). Larvae also show circadian effects in their startle responses following alarm substance exposure (see Section “Alarm (Schreckstoff) Response”) which are abolished by habenula *clock* disruption (Basnakova et al., 2021).

### Microbiome

The microbial status of larvae also influences their anxiety or mood-related behavior. Germ-free (GF) zebrafish exhibited increased locomotor activity and reduced thigmotactic behavior compared to conventionalized (CV) and conventionally-raised (CR) larvae (Davis et al., 2016; Weitekamp et al., 2021). Furthermore, bacterial load was found to be inversely related to locomotor activity (Weitekamp et al., 2021). However, Phelps et al. (2017) reported no difference in thigmotaxis regardless of colonization, despite GF larvae displaying hyperactivity in dark periods by 10 dpf. No locomotor differences were found between CV and CR groups despite compositional differences in the microbiota profiles (Davis et al., 2016). Microbial colonization was also found to modulate stress responses. In GF larvae, expression of genes related to stress response and cortisol levels did not increase after osmotic stress, and microbial colonization within 24 h was able to restore this cortisol response (Davis et al., 2016). Similarly, colonization of axenic larvae by 6 dpf was shown to block hyperactivity (Phelps et al., 2017). *Lactobacillus plantarum* colonization was also found to reduce thigmotactic behavior, though it did not significantly affect cortisol levels in response to stress (Davis et al., 2016). The microbiome has also been reported to regulate social behavior development by shaping basal forebrain development (Bruckner et al., 2020). Hence, the variation between microbial communities across different locations and zebrafish facilities (Roeselers et al., 2011) could also contribute to differences in behavioral endophenotypes observed.

## Open Questions, Challenges, and Future Prospects

Despite its many advantages, there are certainly limitations of the larval zebrafish in modeling mental health endophenotypes. For instance, larval zebrafish may insufficiently capture more complex emotional, social, and cognitive phenomena as compared to adult zebrafish or even rodent or primate models. The zebrafish brain lacks a prefrontal cortex which is heavily involved in reward and emotional processing (Drevets, 2001; Keedwell et al., 2005). In addition, subcortical circuits that are largely involved in socio-emotional behavior and learning, such as the hippocampus and amygdala, may be present but not structurally well-conserved in the zebrafish model (Geng and Peterson, 2019). The midbrain dopaminergic system which is crucial for reward learning and motivational salience (Bissonette and Roesch, 2016) is also lacking in zebrafish (Schweitzer and Driever, 2009; Du et al., 2016). While some learning assays have been developed in larvae (Roberts et al., 2013; Dempsey et al., 2022) (see Sections “Learned Helplessness” and “Age”), larval zebrafish are generally less capable of higher-order cognitive processes such as learning and memory-related tasks (Lovett-Barron, 2021). Hence, the full range of endophenotypes may not be accessible in this model, and complementary models will have to be used in cases where the costs outweigh the benefits.

An important criterion for a candidate endophenotype is that it has an underlying genetic, biochemical or neural circuitry mechanism. However, many of the underlying pathways mechanisms are not exclusive and interact extensively. The HPA axis, including CRH and pituitary corticotrophs, plays a central role in regulating stress levels, sleep, locomotion and dark avoidance (De Marco et al., 2013, 2016; Wagle et al., 2022) in zebrafish. The monoamine class of neurotransmitters, including serotonin, is also involved in avoidance, arousal, and appetitive behavior endophenotypes. Habenula-raphe circuitry has been implicated both in the alarm response and learned helplessness (Lee et al., 2010; Andalman et al., 2019; Basnakova et al., 2021). Feeding, social, and defensive behaviors have been shown to be modulated by oxytocin and other hypothalamic neuropeptides (Wee et al., 2019a,^2022^; Landin et al., 2020; Lovett-Barron et al., 2020). Another neuropeptide, Pth, is involved in both social behavior as well as startle responsiveness (Anneser et al., 2020, 2022). When endophenotypes share an underlying pathway, it could suggest that these behaviors are related. At the same time, two behaviors with distinct pathways can be connected by interactions across these pathways. Brain and organism-wide imaging or transcriptomic approaches (Ahrens et al., 2013; Thyme et al., 2019; Marques et al., 2020), which are growing increasingly prevalent in the field, as well as other novel experimental or computational modeling techniques probing molecular, genetic, and circuit interactions (Vladimirov et al., 2018; Wanner and Vishwanathan, 2018; Tabor et al., 2019; Loring et al., 2020), will be crucial to dissecting these intricate relationships.

Recent studies have also begun to probe the relationships between mental health-related endophenotypes, by conducting different behavioral assays on the same fish or under the same contexts. For example, Blaser and Rosemberg (2012) found that light avoidance and the tank diving response in adult zebrafish are dissociable behavioral traits. In another study, van den Bos et al. (2019) showed dissociation between thigmotaxis and startle responsiveness. We have now shown similar dissociations between light/dark choice and thigmotaxis, and thigmotaxis from light-dark transition behavior or night-time startle in response to acute or prolonged osmotic stress (Cheng et al., 2022).

Further, the relationships between endophenotypes are likely context-dependent and may be influenced by factors such as those reviewed in Section “Controls and Considerations in Endophenotype Investigation.” For example, maternal stress had either correlated or dissociable effects on habituation and thigmotaxis depending on strain background (AB or TL) (van den Bos et al., 2019). In our light/dark choice experiments, salt-induced thigmotaxis was observed during the light/dark but not uniform light period, suggesting an interaction between environmental state and anxiety behavior (Cheng et al., 2022). Increasing salt or dark intensities also reduced the strength of correlations between endophenotypes. Finally, we found that osmotic stress suppressed feeding in a strain-dependent manner (Cheng et al., 2022). Importantly, research into endophenotype correlations, dissociations, and context dependence goes beyond helping us understand endophenotypes better – the convergent pathways underlying such relationships are potential novel drug targets (Kalueff et al., 2014b; Kalueff and Stewart, 2015).

In this review, we focus on behavioral endophenotypes. However, the value of biological endophenotypes in studying mental health cannot be discounted. Changes in heart rate or other cardiac parameters could be indicative of anxiety state. Mild electric shock to cause startle in larval zebrafish caused transient bradycardia while repeated shocks triggered delayed tachycardia (Mann et al., 2010). A sustained increase in heart rate was also induced by a chemical irritant (Roberts et al., 2020). Heart rate can be measured via imaging larvae embedded in agarose (Mann et al., 2010) while more complex features such as blood flow velocity and circulation can be assessed concurrently with behavioral responses (fin beating) using microfluidic set-ups (Subendran et al., 2021). Another biological endophenotype is cortisol levels that can be assayed via radioimmunoassays (Steenbergen et al., 2012) or enzyme-linked immunosorbent assays (ELISAs) (Yeh et al., 2013). The endpoint of HPA axis activation in the stress response is cortisol release. Thus, cortisol levels could be a good proxy for the stress state of the fish and have been used in the validation of behavioral endophenotypes (De Marco et al., 2014, 2016; Bai et al., 2016). However, as Bai et al. (2016) showed, there was again a dissociation between changes in cortisol levels and changes in dark avoidance behavior. When using biological endophenotypes, extra caution must be taken to ensure that changes in these biological markers were the result of changes in mood/anxiety state as opposed to off-target physiological effects. Also, behavioral assays can better distinguish between different aspects and mechanisms of anxiety or mood disorders while biological markers predominantly reflect overall stress or mood levels.

In summary, a nuanced and detailed understanding of the state-dependent relationships between endophenotypes and their underlying genetic, molecular and circuit mechanisms will be a crucial next step in leveraging the zebrafish for mental health modeling.

## Author Contributions

JT wrote the manuscript with the assistance of RA. CW conceived and edited the manuscript. All authors contributed to the article and approved the submitted version.

## Conflict of Interest

The authors declare that the research was conducted in the absence of any commercial or financial relationships that could be construed as a potential conflict of interest.

## Publisher’s Note

All claims expressed in this article are solely those of the authors and do not necessarily represent those of their affiliated organizations, or those of the publisher, the editors and the reviewers. Any product that may be evaluated in this article, or claim that may be made by its manufacturer, is not guaranteed or endorsed by the publisher.
